# Population heterogeneity in developmental trajectories of internalising and externalising mental health symptoms in childhood: differential effects of parenting styles

**DOI:** 10.1017/S2045796023000094

**Published:** 2023-03-31

**Authors:** Ioannis Katsantonis, Jennifer E. Symonds

**Affiliations:** 1 Psychology, Education and Learning Studies Research Group, Faculty of Education, University of Cambridge, 184 Hills Rd, Cambridge CB2 8PQ, UK; 2 School of Education, University College Dublin, Roebuck Offices Belfield, Dublin 4, Ireland

**Keywords:** Externalising symptoms, internalising symptoms, mental health, parenting styles

## Abstract

**Aims:**

Multiple studies have connected parenting styles to children's internalising and externalising mental health symptoms (MHS). However, it is not clear how different parenting styles are jointly influencing the development of children's MHS over the course of childhood. Hence, the differential effects of parenting style on population heterogeneity in the joint developmental trajectories of children's internalising and externalising MHS were examined.

**Method:**

A community sample of 7507 young children (ages 3, 5 and 9) from the *Growing Up in Ireland* cohort study was derived for further analyses. Parallel-process linear growth curve and latent growth mixture modelling were deployed.

**Results:**

The results indicated that the linear growth model was a good approximation of children's MHS development (CFI = 0.99, RMSEA = 0.03). The growth mixture modelling revealed three classes of joint internalising and externalising MHS trajectories (VLMR = 92.51, *p* < 0.01; LMR = 682.19, *p* < 0.01; *E* = 0.86). The majority of the children (83.49%) belonged to a low-risk class best described by a decreasing trajectory of externalising symptoms and a flat low trajectory of internalising MHS. In total, 10.07% of the children belonged to a high-risk class described by high internalising and externalising MHS trajectories, whereas 6.43% of the children were probable members of a mild-risk class with slightly improving yet still elevated trajectories of MHS. Adjusting for socio-demographics, child and parental health, multinomial logistic regressions indicated that hostile parenting was a risk factor for membership in the high-risk (OR = 1.47, 95% CI 1.18–1.85) and mild-risk (OR = 1.57, 95% CI 1.21–2.04) classes. Consistent (OR = 0.75, 95% CI 0.62–0.90) parenting style was a protective factor only against membership in the mild-risk class.

**Conclusions:**

In short, the findings suggest that a non-negligible proportion of the child population is susceptible to being at high risk for developing MHS. Moreover, a smaller proportion of children was improving but still displayed high symptoms of MHS (mild-risk). Furthermore, hostile parenting style is a substantial risk factor for increments in child MHS, whereas consistent parenting can serve as a protective factor in cases of mild-risk. Evidence-based parent training/management programmes may be needed to reduce the risk of developing MHS.

## Introduction

Persuasive evidence suggests that parenting styles significantly influence children's developmental outcomes and, especially, their internalising and externalising mental health symptoms (MHS) (Bayer *et al*., 2006, 2011*b*; Williams *et al*., 2009; Lewis *et al*., 2014; Braza *et al*., 2015). Although research studies have examined the heterogeneity in developmental trajectories of MHS, the differential effects of parenting style on children's MHS trajectories remain largely overlooked. Preceding empirical evidence suggests that children's MHS trajectories are varying significantly from study to study (Fernandez Castelao and Kröner-Herwig, 2014; Parkes *et al*., 2016; Patalay *et al*., 2016, 2017; Vaillancourt *et al*., 2017; Flouri *et al*., 2018; Papachristou and Flouri, 2020), however, most of these studies did not consider parenting styles collectively as predictors of children's trajectory profiles of internalising and externalising MHS.

### Heterogeneous developmental trajectories of MHS

Previous research has investigated the developmental trajectories of children's MHS from a person-centred perspective by identifying heterogeneous subpopulations of children with specific characteristics in their MHS development. Most of the studies have usually found that the majority of children's MHS development was exhibiting a stable low trajectory of internalising and/or externalising MHS, in addition to multiple other trajectories that usually described stably increasing or moderate-increasing/decreasing trajectories of internalising and externalising MHS (Patalay *et al*., 2015, 2017; Parkes *et al*., 2016). Specific trends are emerging from these studies suggesting the existence of more than two distinct developmental trajectories (Miner and Clarke-Stewart, 2008; Parkes *et al*., 2016; Patalay *et al*., 2016; Vaillancourt *et al*., 2017; Flouri *et al*., 2018; Papachristou and Flouri, 2020).

It should be noted that the heterogeneity of the developmental trajectories of MHS may occur due to various reasons. Amongst these reasons may be the mixtures of different developmental periods, the nature of the sampling, the different analytic techniques, the socio-demographic composition of the samples and the measure of MHS used. In the present study, we utilised a national cohort sample of Irish children that covered a range of social-economic backgrounds. Hence, it was expected that some discrepancies between studies would emerge.

Nevertheless, a limitation of some preceding empirical works is that they examined internalising and externalising symptoms separately (Miner and Clarke-Stewart, 2008; Fernandez Castelao and Kröner-Herwig, 2014; Patalay *et al*., 2015; Parkes *et al*., 2016). Research studies have noted that internalising and externalising MHS display some comorbidity where a common trait underlies exposure to both domains of psychopathology (Willner *et al*., 2016). Evidence coming from behaviour genetics (Cosgrove *et al*., 2011; Lahey *et al*., 2011) and survey (Goodman *et al*., 2010; D'Urso and Symonds, 2022; Katsantonis, 2022) research suggests that internalising and externalising MHS are strongly correlated. Hence, we aimed to identify common MHS subtypes of internalising and externalising developmental trajectories in order to examine the degree of comorbidity between these broad types of MHS.

### Parenting styles predicting developmental trajectories of MHS

Parenting styles are very important for children's typical development since they play a major role in creating an affective climate that serves as the foundation of the parent–child interactions (Darling and Steinberg, 1993). They are connected to multiple developmental outcomes, and can be either a risk or a protective factor for children's MHS (Bayer *et al*., 2006, 2011*b*; Lewis *et al*., 2014; Braza *et al*., 2015). Thus, rightly the UNICEF refers to parenting as fundamental for child mental health (United Nations Children's Fund, 2021).

Different parenting styles have been connected to children's MHS in different ways. For example, warm or warm-engaged parenting style was found to be a significant predictor of reduced child MHS (Buschgens *et al*., 2010; Bayer *et al*., 2011*b*; Parkes *et al*., 2016), whilst over-involved/protective, harsh and disciplinary parenting are predictors of increased MHS (Bayer *et al*., 2011*b*; Lewis *et al*., 2014). Similarly, studies have found that hostility was linked with a constellation of affective and behavioural MHS in toddlers (Dwairy, 2008; Bayer *et al*., 2011*b*; Schwerdtfeger *et al*., 2013). Hence, depending on the frequency and the nature of the parenting style, it may be either a substantial risk or protective factor for the development of children's MHS.

Although different types of parenting styles have been linked with greater or lower likelihood for development of internalising and externalising MHS, it is striking that few studies have estimated the differential effects of parenting styles on children's heterogeneous developmental trajectories of MHS. For example, Weeks *et al.* (2014) found that hostile parenting style predicted membership in the onset trajectory of internalising MHS compared to the low-stable trajectory. Likewise, Parkes *et al.* (2016) reported that warm parenting style predicted low likelihood of membership in the trajectory of high and decreasing internalising MHS compared to the stable-low trajectory. Since different parenting styles may co-occur in the same parent (Heberle *et al*., 2015), it is important to collectively explore the impact of various parenting styles on children's MHS development.

#### Aims and hypotheses

Some evidence gaps were identified regarding the differential effects of parenting styles on children's developmental trajectories of MHS. Specifically, previous studies have not thoroughly examined the effects of parenting styles on the population heterogeneity in MHS trajectories across childhood. Additionally, many preceding studies examined the trajectories of internalising and externalising MHS separately or without considering how the trajectories of internalising MHS may influence the trajectories of externalising MHS, and vice versa. Thus, we aimed to address these gaps by deploying a person-centred approach (Muthén and Muthén, 2000) along with robust national longitudinal data. Hence, the following hypotheses guided the present study. It was hypothesised that the analysis of heterogeneity in children's joint MHS trajectories would reveal more than two distinct developmental trajectories (H1). Further, it was expected that the developmental trajectories of internalising and externalising MHS would be correlated (H2). Finally, it was hypothesised that, adjusting for covariates, warm and consistent parenting styles would predict membership in the subpopulations with decreasing trajectories (H3), whilst hostile parenting style would be linked with subpopulations of accelerating trajectories (H4).

## Method

### Sample and dataset

Data come from three of the main waves of the *Growing Up in Ireland* infant cohort study (McNamara *et al*., 2020). The cohort study had five waves of data available. At 9 months old, 11 134 children and their families participated, whilst 9793 children (88% response rate) participated at age 3 (wave 2). In total, 9001 children (80.8%) participated in wave 3, whereas 8032 children participated in wave 5 (72.1%). Although another wave of data is available at age 7/8, this wave's data collection was conducted through a postal questionnaire and the response rate was 48% (McNamara *et al*., 2020). Since the sampling weights do not permit representative analysis of all the waves (Quail *et al*., 2019), the longitudinally matched sample size amounted to 7507 children aged 3 years old (wave 2) that were followed at age 5 (wave 3) and at age 9 (wave 5).

### Measures

#### Internalising and externalising MHS

The four negative symptom scales from the Strengths and Difficulties Questionnaire (SDQ) (Goodman, 1997, 2001; Goodman *et al*., 2000) were administered to the cohort. Parent-reported (>98% mothers) scores were used in this study. Despite that the SDQ is a broad screening measure of MHS, the scales show good sensitivity, specificity (Cocker *et al*., 2018) and accuracy in predicting common mental disorders (Goodman *et al*., 2000; Goodman and Goodman, 2011). According to the SDQ validation studies, the summed composite of the emotional symptoms and peer problems scales forms the internalising symptoms score, whilst the composite of the conduct problems and hyperactivity scales forms the externalising score (Goodman *et al*., 2010). All composite scores range from 1 to 10. The scales displayed good reliability across the survey's waves (McCrory *et al*., 2013; McNamara *et al*., 2020).

#### Parenting styles

Three scales adopted from the Longitudinal Study of Australian Children (LSAC) were indexing parenting styles (McNamara *et al*., 2020). Composite scores on warm (six items – display of affection, awareness of child's needs), consistent (five items – consistent expectations and applications of rules) and hostile (six items – overcontrolling, discipline) parenting styles ranging from 1 to 5 were available. The scales displayed good reliability across the survey's waves (McCrory *et al*., 2013; McNamara *et al*., 2020). Parenting styles from wave 2 were included in the modelling.

#### Covariates

Drawing upon a developmental and psychopathology multisystem resilience approach (Masten *et al*., 2021), three types of covariates, namely socio-demographics, child health and parental health, from wave 2 (see Table 1) were included in the modelling since they have been identified either as risk or protective factors.

**Table 1.** Sample descriptive statistics for all cohort members

Variable		Descriptive statistic
Household annual income (ref: lowest quintile)		
2nd quintile	%	18.08
3rd quintile	%	20.22
4th quintile	%	21.21
Highest quintile	%	23.64
Sex (ref: males)		
Females	%	49.66
Household structure (ref: two parents)		
Lone parent	%	10.22
Parental ethnicity (ref: ethnic minority)		
Ethnic majority (Irish)	%	84.25
Parental employment (ref: unemployed)		
Work/training	%	58.83
School/education	%	1.61
Home duties	%	32.48
Other	%	2.32
Highest parental qualification (ref: non-degree)		
Degree level or higher parental qualification	%	42.01
Child's longstanding illness/condition/disability (ref: no)		
Yes	%	15.18
Child's low general health	M (SD)	1.27 (0.49)
Parental on-going physical/mental health problem (ref: no)		
Yes	%	13.83
Parental general health	M (SD)	2.03 (0.91)
Parental stress	M (SD)	12.25 (4.10)
Internalising score		
Internalising age 3	M (SD)	2.45 (2.16)
Internalising age 5	M (SD)	2.45 (2.39)
Internalising age 9	M (SD)	2.96 (2.89)
Externalising score		
Externalising age 3	M (SD)	5.14 (3.28)
Externalising age 5	M (SD)	4.67 (3.33)
Externalising age 9	M (SD)	4.16 (3.39)
Parenting styles		
Warm parenting style	M (SD)	4.74 (0.37)
Hostile parenting style	M (SD)	1.79 (0.48)
Consistent parenting style	M (SD)	4.02 (0.71)

*Note*: ref, reference category; data were parent-reported by the primary caregiver (>0.98% mothers).

### Analysis strategy

Preliminary data management was conducted through Stata 16 (StataCorp., 2019), whilst latent variable modelling and multinomial regressions were conducted with M*plus* 8.7 (Muthén and Muthén, 2017). A two-step strategy was followed in the modelling. In the first instance, we estimated a linear parallel-process growth model (LGM) with correlated two intercepts and two growth factors defined by the composite internalising and externalising MHS scores. Within-wave residual correlations were estimated per the suggestions of the modification indices. After evaluating the fit of the model, we opted for a person-centred approach to identify population heterogeneity in developmental trajectories of MHS. To this end, a parallel-process growth mixture model (GMM) with equal variances (the M*plus* default) across classes was specified and estimated (Muthén and Muthén, 2000; Jung and Wickrama, 2008). Missing data were handled with full-information maximum likelihood (Enders, 2022). After determining the number of latent classes, the most probable class membership was extracted and multinomial regressions were estimated to examine the effects of parenting styles, whilst controlling for the effects of covariates. The longitudinal sampling weight (including non-response adjustment) at age 9 was applied to all inferential analyses.

All models were estimated with the robust maximum likelihood estimator. To evaluate the fit of the LGM, the conventional thresholds in goodness-of-fit indices were utilised. Specifically, values above/close to 0.95 in the Comparative Fit Index (CFI) and Tucker-Lewis Index (TLI) in conjunction with values less than 0.06 and 0.08 in the Root Mean Square Error of Approximation (RMSEA) and the Standardised Root Mean Residual (SRMR), respectively, are considered indicators of good fit to the sample covariance matrix (Hu and Bentler, 1999). To decide on the number of latent classes, we considered a combination of fit indices in GMM, and the clinical and practical interpretability of the classes. Specifically, we consulted the entropy, the Akaike Information Criterion (AIC), the Bayesian Information Criterion (BIC), the sample-adjusted Bayesian Information Criterion (sBIC), the Vuong–Lo–Mendel–Rubin (VLMR) and the adjusted Lo–Mendel–Rubin (LMR) likelihood tests. Entropy values above 0.8 are preferred, whereas the AIC and (s)BIC values are used to compare two nested models for parsimony (Nylund *et al*., 2007). Given that the BIC is known to persist decreasing with additional classes, the BIC values were plotted to identify the point (‘elbow’) of diminishing returns (Nylund-Gibson and Choi, 2018). Finally, the VLMR and LMR are likelihood ratio tests that compare the model with *n*–1 classes with the model with *n* classes and provide *p*-values. Non-significant likelihood ratio tests indicate that the model with *n* classes does not offer an improvement in model-data fit compared to the *n*–1 classes model (Nylund *et al*., 2007; Ferguson *et al*., 2020). To ascertain the extent to which the correct number of classes was identified and the best loglikelihood replicated, a large number of 500 random starting values was utilised and more latent classes were extracted.

## Results

Sample descriptive statistics, such as means, SDs, or percentages, for key outcomes and covariates are presented in Table 1.

As a reference only, the sample was distributed into the age-appropriate cut-off centiles based on the new fourfold classification of the scores on the SDQ scales in the UK (www.SDQinfo.org). As can be seen in Table 2, most children's scores on all SDQ scales were close to average with fewer children scoring in the higher centiles.

**Table 2.** Distribution of the cohort sample in each centile cut-off of mental health symptoms based on the SDQ fourfold classification

	Total *N*^a^	*N* per centile			
Age 3		80%	92%	96%	99.99%
Emotional problems	7504	6259	687	327	231
Conduct problems	7503	5953	753	458	339
Hyperactivity	7502	6535	447	261	259
Peer problems	7504	6376	616	305	207
Age 5		80%	90%	95%	99.99%
Emotional problems	7505	6600	450	369	86
Conduct problems	7505	5929	879	597	100
Hyperactivity	7504	6216	784	248	256
Peer problems	7504	6669	443	219	173
Age 9		80%	90%	95%	99.99%
Emotional problems	7498	6000	638	597	263
Conduct problems	7499	6440	576	412	71
Hyperactivity	7499	6222	717	221	339
Peer problems	7499	6568	419	252	260

a Listwise sample size; 80% centile: close to average; 90/92% centile: slightly raised; 95/96% centile: high; 99.99% centile: very high; age-appropriate cut-offs were derived from www.SDQinfo.org.

### Heterogeneity in MHS trajectories

An intercept-only model was found to be degraded compared to the intercept-slope model, Satorra–Bentler *χ*^2^(9) = 926.16, *p* < 0.001. The linear parallel-process LGM (intercept-slope) exhibited great fit to the data, i.e. scaled *χ*^2^(4) = 30.22, *p* < 0.001, CFI = 0.996, TLI = 0.98, RMSEA = 0.03, SRMR = 0.013. Thus, we could be confident that a linear model of change could be a good approximation for the MHS trajectories. The LGM parameters are presented in the Supplementary materials. Next, we estimated the GMM to identify possible heterogeneity in the trajectories of MHS. The model with three classes of MHS trajectories exhibited significant improvement compared to the two-class model. Moreover, the likelihood ratio tests revealed that the addition of a fourth class did not result in improved model-data fit (*p* > 0.05). The ‘elbow’ plot of the BIC values showed that adding more than three classes did not improve fit substantially (see Supplementary materials). Additionally, we estimated further models where the variances of the intercept and slope parameters were freely varying between classes. However, the variance–covariance matrices were not positive definite, and thus, the variances were held equal across classes. Keeping in mind that this was a general population sample, we did not expect extreme variation in mental health symptomatology, and thus, the three-class model was retained. Fit indices for the models are presented in Table 3.

**Table 3.** Fit indices for the parallel-process growth mixture models

*N* classes	AIC	BIC	sBIC	Entropy (E)	VLMR	Adjusted LMR
1	213 329.75	213 488.99	213 415.90			
2	211 696.48	211 890.34	211 801.37	0.87	85.92***	1607.243***
3	211 009.00	211 237.48	211 132.61	0.86	92.51**	682.19**
4	210 566.16	210 829.25	210 708.50	0.86	173.07	442.91
5	210 090.89	210 388.60	210 251.96	0.83	183.22	464.41

*Note*: ***p < 0.001; **p < 0.01; the bootstrap LRT is not available with sampling weights.

**Fig. 1.**
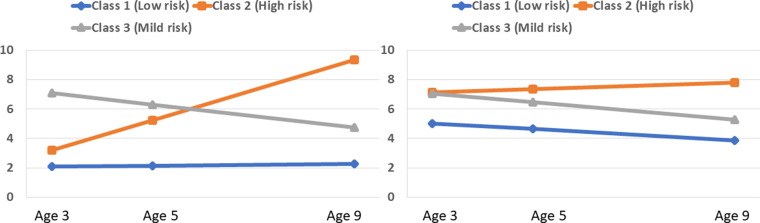
Estimated internalising (left) and externalising (right) MHS trajectories.

The model revealed that 83.49% (*N* = 6268) of the children belonged to class 1 (low risk) that was characterised by a flat low trajectory of internalising MHS, and a declining trajectory of externalising MHS. In contrast, 10.07% (*N* = 756) of the children were members of a high-risk class 2 that was characterised by an accelerating high trajectory of internalising and externalising MHS. Finally, 6.43% (*N* = 483) of the children belonged to class 3 with MHS trajectories that were slightly improving but still elevated (mild-risk). The estimated trajectories are shown in Fig. 1.

The growth parameters are presented in Table 4. The internalising intercept was negatively associated with the slope of externalising MHS, *r* = −0.15, *p* < 0.01. Higher externalising MHS at age 3 were associated with a decelerating externalising MHS development, *r* = −0.28, *p* < 0.001. In contrast, children's internalising scores underwent a substantial shift between ages 3 and 9, *r* = 0.09, *p* > 0.05. The externalising and internalising intercepts were positively associated indicating that children with higher initial status in internalising MHS exhibited high initial status in externalising MHS, *r* = 0.49, *p* < 0.001. Finally, the two slopes were also positively correlated, *r* = 0.46, *p* < 0.001, indicating that higher internalising trajectories were associated with higher externalising trajectories.

**Table 4.** Growth parameters for the parallel-process growth mixture model

Parameter	Class 1: low risk		Class 2: high risk		Class 3: mild risk	
	INT (1)	EXT (2)	INT (1)	EXT (2)	INT (1)	EXT (2)
Intercept *μ*	2.07***	5.03***	3.19***	7.15***	7.07***	7.04***
Slope *μ*	0.03**	−0.19***	1.03***	0.11	−0.39***	−0.29***
Intercept *σ*^2^	1.01***	6.45***	1.01***	6.45***	1.01***	6.45***
Slope *σ*^2^	0.02	0.21***	0.02	0.21***	0.02	0.21***
Correlations (*r*) between growth parameters						
*r* (intercept 1, slope 1)	0.09					
*r* (intercept 2, slope 2)	−0.28***					
*r* (intercept 1, slope 2)	−0.15**					
*r* (intercept 2, slope 1)	0.05					
*r* (intercept 1, intercept 2)	0.49***					
*r* (slope 1, slope 2)	0.46**					

*Note*: ***p < 0.001; **p < 0.01; INT, internalising; EXT, externalising; equal *σ*^2^ across classes.

### Effects of parenting styles on MHS classification

Having established three heterogeneous trajectories of MHS, the effects of the three parenting styles on children's MHS trajectories were estimated using multinomial logistic regressions adjusting for the covariates. To ascertain the extent to which multicollinearity may have occurred, the variance inflation factors (VIF) were inspected and found to be less than 6 (mean VIF = 1.84). Thus, no extreme collinearity was diagnosed for the model. The results of the multinomial logistic regressions are presented in Table 5.

**Table 5.** Multinomial logistic regressions predicting high-risk class 2 and mild-risk class 3 membership *v.* low-risk class 1 membership

	High-risk		Mild-risk	
	OR	95% CI	OR	95% CI
Predictor				
*Socio-demographics*				
Child sex (ref: male)				
Child sex (female)	**1**.**31**	**1.05; 1.63**	1.13	0.87; 1.47
Household structure (ref: two-parents)				
Household structure (lone parent)	**1**.**42**	**1.04; 1.94**	**1**.**54**	**1.04; 2.28**
Income (ref: lowest quintile)				
Income (2nd quintile)	0.86	0.62; 1.18	1.06	0.69; 1.63
Income (3rd quintile)	0.71	0.49; 1.02	1.05	0.69; 1.62
Income (4th quintile)	0.67	0.45; 1.00	0.84	0.53; 1.33
Income (5th quintile)	**0**.**63**	**0.42; 0.95**	0.70	0.41; 1.18
Ethnicity (ref: ethnic minority)				
Ethnic majority (Irish)	1.24	0.91; 1.68	0.96	0.70; 1.31
Employment (ref: unemployed)				
Employment (school/education)	1.00	0.45; 2.22	**0**.**04**	**0.00; 0.34**
Employment (work/training)	0.73	0.46; 1.15	0.87	0.47; 1.58
Employment (home duties)	0.78	0.50; 1.22	1.12	0.62; 2.01
Employment (other)	0.83	0.40; 1.72	0.79	0.28; 2.19
Highest parental qualification (ref: no degree-level)				
Highest parental qualification (degree-level or higher)	0.85	0.66; 1.08	0.95	0.72; 1.26
*Child's health*				
Low general health	**1**.**32**	**1.05; 1.65**	**1**.**80**	**1.40; 2.33**
Longstanding illness/condition/disability	**1**.**56**	**1.15; 2.12**	1.32	0.92; 1.88
*Parental health*				
Low general health	1.12	0.99; 1.28	**1**.**19**	**1.01; 1.39**
On-going physical/mental health problem	**1**.**46**	**1.10; 1.95**	0.93	0.64; 1.34
Parental stress	**1**.**09**	**1.06; 1.12**	**1**.**08**	**1.04; 1.12**
*Parenting styles*				
Warm style	1.30	0.96; 1.75	0.74	0.55; 1.01
Hostile style	**1**.**47**	**1.18; 1.85**	**1**.**57**	**1.21; 2.04**
Consistent style	0.87	0.74; 1.02	**0**.**75**	**0.62; 0.90**

*Note*: ref, reference category; OR, odds ratio; 95% CI, 95% confidence interval; *N* = 7507; values in bold reached statistical significance according to the 95% CI. Statistical significance did not vary between the logit and OR coefficients.

Hostile parenting style was a substantial predictor of membership in the high-risk class (OR = 1.47, 95% CI 1.18–1.85) and the mild-risk class (OR = 1.57, 95% CI 1.21–2.04). Warm parenting style did not predict membership in the high-risk (OR = 1.30, 95% CI 0.96–1.75) or the mild-risk (OR = 0.74, 95% CI 0.55–1.01) classes. Although consistent parenting style did not predict membership in the high-risk class (OR = 0.87, 95% CI 0.74–1.02), it was a protective factor against membership in the mild-risk class (OR = 0.75, 95% CI 0.62–0.90). The results for the covariates are discussed in the Supplementary materials.

## Discussion

The person-centred approach revealed some extent of heterogeneity in children's MHS trajectories. Contrary to most preceding evidence (Patalay *et al*., 2015, 2016, 2017; Vaillancourt *et al*., 2017; Flouri *et al*., 2018; Papachristou and Flouri, 2020), only three joint trajectories of internalising and externalising MHS were identified. Thus, our H1 was retained. In other words, despite a substantial number of previous studies indicating more than three distinct classes of MHS trajectories, the present modelling identified that 10.07% of the children followed a MHS developmental trajectory that was best described as high-risk with characteristics of accelerating high MHS trajectories. Another smaller class of children (6.43%) was characterised by improving yet still elevated trajectories of MHS, and was, thus, defined as mild-risk class. In short, most Irish children (83.49%) did not seem to be at-risk of developing some MHS. The majority of the Irish children's MHS development was following a decreasing externalising trajectory and a stable flat-low internalising trajectory. These findings seem to agree with psychiatric epidemiological evidence suggesting decreasing trends in children's internalising and externalising MHS (Maughan *et al*., 2008; Sellers *et al*., 2015; Pitchforth *et al*., 2019).

As mentioned, most of the preceding evidence did not examine (Miner and Clarke-Stewart, 2008; Fernandez Castelao and Kröner-Herwig, 2014; Patalay *et al*., 2015; Parkes *et al*., 2016) or discuss in depth the correlated nature of the MHS trajectories. Previous studies have reported inconclusive results regarding the correlated nature of the internalising and externalising MHS with some studies reporting low or non-significant correlations (Rhee *et al*., 2015), whereas other studies showed significant comorbidity across time (Willner *et al*., 2016). Hence, in the current study, we clarified this through a developmental perspective by exploring the association between the growth parameters of the internalising and externalising trajectories. This resulted in a longitudinal estimate of a moderate degree of comorbidity between the development of these broad MHS in this community sample. Thus, H2 was confirmed.

Last but not least, the present study sought to evaluate the impact of parenting styles on the heterogeneity in children's MHS developmental trajectories. Using a developmental and psychopathology multisystem resilience approach (Masten *et al*., 2021), we controlled for various other variables that have been linked with children's (mal-)adaptive functioning. After adjusting for several socio-demographic factors and child and parental health, the multinomial regressions illustrated that hostile parenting was a significant risk factor for the development of MHS. In précis, the model revealed that hostile parenting was predicting greater odds of membership in the high- and mild-risk classes of MHS trajectories. These findings are congruent with extant research that found a significant longitudinal effect of hostility and consistency on children's MHS (Bayer *et al*., 2011*a*; Lewis *et al*., 2014; Parkes *et al*., 2016). In contrast, consistent parenting style was a protective factor only against being at mild-risk for developing MHS. Despite evidence suggesting a protective effect of warm parenting style on children's MHS development (Buschgens *et al*., 2010; Bayer *et al*., 2011*b*; Parkes *et al*., 2016), its longitudinal effect on MHS was not significant in our modelling. Thus, H3 was partially rejected and H4 was confirmed. The differential contributions of the parenting styles cement the theoretical tenant that several parenting styles may co-exist in a single family (Heberle *et al*., 2015). To the best of our knowledge, no previous study has examined collectively the effects of warm, consistent and hostile parenting styles on children's heterogeneous profiles of MHS trajectories. Regarding the covariates, in both the high- and mild-risk classes of MHS, we found that children's and parental general health, as well as the household structure and parental stress were consistently non-negligible risk factors for MHS. Being a female child predicted greater odds of membership in the high-risk class, whereas higher income was a protective factor against high-risk MHS confirming the social gradient in MHS.

Overall, the present study is distinct from preceding works (Fernandez Castelao and Kröner-Herwig, 2014; Patalay *et al*., 2017; Flouri *et al*., 2018; Papachristou and Flouri, 2020) in the sense that it focused only on the developmental period of childhood. This allowed us to identify distinct classes of MHS developmental trajectories that were influenced only by the developmental characteristics of childhood. Additionally, a substantial part of previous evidence was based on cohort studies in the UK (Parkes *et al*., 2016; Patalay *et al*., 2017; Flouri *et al*., 2018; Papachristou and Flouri, 2020), which does not necessarily translate to other contexts. Finally, the present study's results may differ from preceding evidence given the analytic choice of a parallel-process modelling in contrast to previous approaches that modelled internalising and externalising MHS separately as distinct broad types of MHS (Fernandez Castelao and Kröner-Herwig, 2014; Patalay *et al*., 2015, 2016; Parkes *et al*., 2016).

Nevertheless, the present work also had some limitations. Specifically, it should be underscored that SDQ is a broad screening tool of children and adolescents’ MHS and does not necessarily target specific symptoms of the most common mental disorders. Moreover, evidence suggests that SDQ may not have high criterion validity in predicting emotional and behavioural disorders (Aydin *et al*., 2022). Furthermore, given the limitations of the data at hand we did not have any further indicators of other parenting styles. Additionally, without more waves of data, it was not possible to evaluate models of non-linear growth. Finally, given that the data came exclusively from parent reports/interviews, this may suggest that social desirability may bias somewhat the results, especially, concerning the parenting styles.

### Implications for policy and practice

The present findings may have significant implications for policy and practice. For instance, the correlated nature of the developmental trajectories of internalising and externalising MHS indicates that mental health professionals should always screen for both types of MHS. Furthermore, the person-centred modelling indicated that only a small proportion of the general population of young children may be at-risk for increased MHS. However, mental health practitioners should be vigilant to identify those children. Thus, more robust extensive screening may be needed. Most importantly, the present study's results underscored the importance of parenting styles for the identification of children at-risk for developing high/mild MHS. Therefore, we suggest that routine background checks of parenting behaviours should be applied while screening children for possible MHS. Given that hostile parenting style was associated with children's membership in the high- and mild-risk classes with high MHS trajectories, we recommend evidence-based parent training/management programmes to improve positive interactions between parent and child.

## Data Availability

The Growing Up in Ireland infant cohort datasets, which were utilised in the present study, were accessed and are available via the Irish Social Science Data Archive – www.ucd.ie/issda. The authors have access to anonymised microdata files which include composite variables only.

## Supplementary material

For supplementary material accompanying this paper visit http://doi.org/10.1017/S2045796023000094. click here to view supplementary material
