# Supplementary material for: Population heterogeneity in developmental trajectories of internalising and externalising mental health symptoms in childhood: differential effects of parenting styles
Source: Epidemiol Psychiatr Sci. 2023 Mar 31;32:e16. doi: 10.1017/S2045796023000094 (PMC10130732; doi:10.1017/S2045796023000094)
Supplement: Supplementary file 1 [file S2045796023000094sup001.docx]

Supplementary Materials EPS

“Population Heterogeneity in Developmental Trajectories of Internalising and Externalising Mental Health Symptoms in Childhood: Differential Effects of Parenting Styles”

**Confirmatory Factor Analysis (CFA) of Internalising and Externalising Composites**

The correlated two-factor CFA model of internalising and externalising MHS based on the composite indicators at age 3 had exceptional fit, scaled χ^2^ (1)=3.11, *p*=0.08, CFI=1.00, RMSEA=0.02. The CFA model at age 5 was also fitting the data very well, scaled χ^2^ (1)= 2.54, *p*=0.11, CFI=1.00, RMSEA=0.01. Similar were the results for the CFA model at age 9, χ^2^ (1)= 4.50, *p*=0.03, CFI=1.00, RMSEA=0.02. The latent factor loadings were substantially above 0.30 across occasions.

**Linear Latent Growth Curve Model (LGM)**

For a detailed description of the LGM specification see inside the analytic strategy section.

Standardised Parameter Estimates for the LGM (whole cohort sample, N=7507)

| Parameter | Parameter value |
| --- | --- |
| Internalising (INT) Factor Loadings (λ) |  |
| Intercept age 3 | .741*** |
| Intercept age 5 | .651*** |
| Intercept age 9 | .536*** |
| Slope age 3 | .000 |
| Slope age 5 | .271*** |
| Slope age 9 | .669*** |
| Externalising (EXT) Factor Loadings (λ) |  |
| Intercept age 3 | .782*** |
| Intercept age 5 | .765*** |
| Intercept age 9 | .755*** |
| Slope age 3 | .000 |
| Slope age 5 | .263*** |
| Slope age 9 | .779*** |
| Latent Growth Parameter Correlations |  |
| s1 WITH i1 | -.086 |
| s2 WITH i2 | -.224*** |
| i1 WITH i2 | .468*** |
| s1 WITH i2 | .178*** |
| s2 WITH i2 | -.096** |
| s2 WITH s1 | .333*** |
| Within-wave Residual Correlations |  |
| INT1 WITH EXT1 | .151*** |
| INT2 WITH EXT2 | .331* |
| INT3 WITH EXT3 | .207*** |
| Latent Growth Parameter Means |  |
| I1 | 1.551*** |
| S1 | .315*** |
| I2 | 2.035*** |
| S2 | -.367*** |
| Latent Growth Parameters’ Variances† |  |
| I1 | 2.738*** |
| S1 | .119*** |
| I2 | 7.067*** |
| S2 | .209*** |

^Note: s1: slope of internalising; i1: intercept of internalising; s2: slope of externalising; i2: intercept of externalising; WITH denotes correlation between two parameters; †Standardised latent growth parameter variances fixed at 1 by default for model identification purposes; ***^*^p^*^<.001; **^*^p^*^<.011; *^*^p^*^<.05^


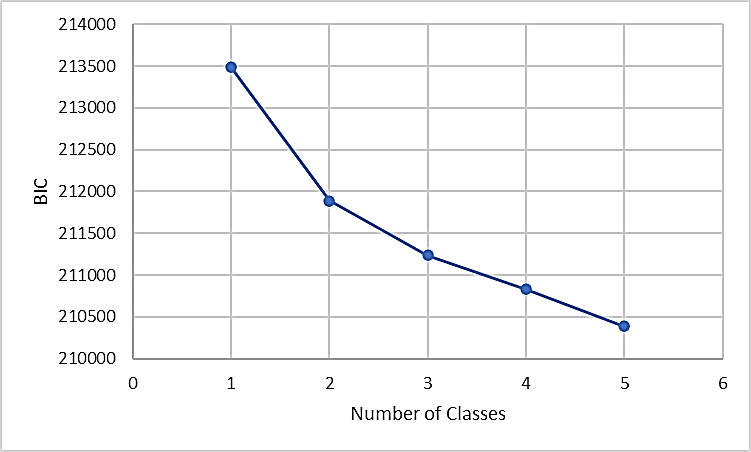


**Figure. ‘Elbow’ plot of BIC values for selecting number of latent classes in growth mixture modelling**

**Results of Multinomial Regressions for Covariates**

Amongst the three clusters of covariates, child and parental general health were identified as the most substantial risk factors. Specifically, children’s low general health was associated with increased likelihood of belonging to the high-risk class (OR=1.32, 95% CI 1.05; 1.65), and the mild-risk class (OR=1.80, 95% CI 1.40- 2.33). Children’s longstanding health condition was also associated with higher likelihood of being at high-risk (OR=1.56, 95% CI 1.15- 2.12). Furthermore, low parental general health only predicted membership in the mild-risk class (OR=1.19, 95% CI 1.01- 1.39). Similarly, inflated levels of parental stress were also related to higher odds of belonging to the high-risk (OR=1.09, 95% CI 1.06- 1.12) and the mild-risk (OR=1.08, 95% CI 1.04- 1.12) classes. Parental on-going health issues were also predicting higher likelihood of the child being at high risk (OR=1.46, 95% CI 1.10- 1.95).

With regards to the socio-demographic profiles, it can be seen that being a female child (OR=1.31, 95% CI 1.05- 1.63) was linked with higher odds of being at high-risk. Similarly, being in a lone-parent household was connected to higher odds of belonging to the high-risk (OR=1.42, 95% CI 1.04- 1.94) and mild-risk (OR=1.54, 95% CI 1.04- 2.28) classes. We did not find evidence suggesting that being from an ethnic majority background predicted children’s classification into high- or mild-risk.

In general terms, we did not find evidence indicating that the parent’s employment status had a differential impact on children’s classification of MHS with the only exception of the parent being in education, which negatively predicted mild-risk membership (OR=0.04, 95% CI 0.00- 0.34). The highest household income quintile was linked with lower odds of belonging to the high-risk class (OR=0.63, 95% CI 0.42- 0.95), indicating a social gradient in MHS.
